# Novel antiviral activity of neuraminidase inhibitors against an avian influenza a virus

**DOI:** 10.1186/1743-422X-8-411

**Published:** 2011-08-19

**Authors:** Hiroshi Ushirogawa, Masanobu Ohuchi

**Affiliations:** 1 Department of Microbiology, Kawasaki Medical School, 577 Matsushima, Kurashiki, Okayama 701-0192, Japan

## Abstract

**Background:**

Neuraminidase (NA) inhibitors used for influenza therapy are believed to prevent the release of progeny virus from the surface of an infected cell. In this study, we found that NA inhibitors have a novel antiviral function against an avian influenza virus.

**Results:**

Madin-Darby canine kidney cells, commonly used for the isolation and propagation of the influenza virus, were infected with an avian influenza viral strain A/chicken/German/N/49(H10N7) (H10/chicken) or a human influenza viral strain A/Osaka/981/98(H3N2) (H3/Osaka) virus. Cells were incubated in a medium without or with a NA inhibitor, oseltamivir carboxylate (GS4071), from 1 to 13 h post infection (p.i.). Infected cells were washed 12 h p.i. to remove GS4071, incubated for 1 h without GS4071, and assayed for virus production. Incubation with GS4071 decreased the production of infectious viruses. When H10/chicken virus-infected cells were incubated with GS4071 from 12 to 13 h p.i. (i.e., 1 h before the virus production assay), the inhibitory effect was clearly observed, however, the same was not evident for H3/Osaka virus-infected cells. Furthermore, viral protein synthesis in infected cells was not affected by GS4071. Using a scanning electron microscope, many single spherical buds were observed on the surface of H3/Osaka virus-infected cells incubated without GS4071, whereas many aggregated particles were observed on the surface of cells incubated with GS4071. However, many long tubular virus-like structures, with no aggregated particles, were observed on the surface of H10/chicken virus-infected cells incubated with GS4071. The same results were obtained when another NA inhibitor, zanamivir, was used.

**Conclusions:**

These results indicate that NA inhibitors interfered with virus particle formation in the H10/chicken virus-infected cells, in which the inhibitor caused the formation of long tubular virus-like structures instead of spherical virus particles.

## Background

Influenza A and B viruses possess two surface spike glycoproteins, hemagglutinin (HA) and neuraminidase (NA). HA mediates binding of the virus to sialoglycan, the receptor of the influenza virus, and fusion of the viral envelope with the cellular endosomal membrane. Thus, HA helps the virus enter target cells. The function of NA is to destroy viral receptors by removing sialic acid residues from sialoglycans, thereby contributing to the release of progeny viruses from infected cells [1,2]. Thus, NA inhibitors are believed to block the release of progeny viruses and interfere with infection.

In addition, several other attributes of NA have been reported. First, NA is essential for several strains to demonstrate their hemagglutinating activity [3,4]; second, NA enhances infection efficiency [5,6]; and third, NA promotes the viral protein synthesis efficiency in cells infected with avian influenza viruses [7]. Thus, NA is a multifunctional protein for influenza virus infection, and hence, NA inhibitors would inhibit the abovementioned functions of NA.

In this study, we discovered that a NA inhibitor prevented virus particle formation under conditions in which the inhibitor does not affect any of the abovementioned functions, which is a novel antiviral function of the NA inhibitor. An inhibitory effect was observed in the cells infected with an avian viral strain, A/chicken/Germany/N/49(H10N7) (H10/chicken). This study suggests that viral NA has the potential to assist virus particle formation at the final stage of viral replication.

## Results

### Effect of the NA inhibitor on the production of infectious viruses

Confluent monolayer cultures of Madin-Darby canine kidney (MDCK) cells were inoculated with H10/chicken or human influenza A/Osaka/981/98(H3N2) (H3/Osaka) virus at a multiplicity of infection (MOI) of 0.3 plaque forming units (pfu) per cell. At 1 h post infection (p.i.), cultures were washed twice with Dulbecco's modified minimum essential medium (DMEM) and incubated in DMEM with or without 2 μM of oseltamivir carboxylate (GS4071) from 1 to 13 h p.i. The 50% inhibitory concentration of GS4071 against NA activity was almost the same between H10/chicken and H3/Osaka viruses, and NA activity of both viruses was completely suppressed by 2 μM GS4071 (data not shown).

**Figure 1:**
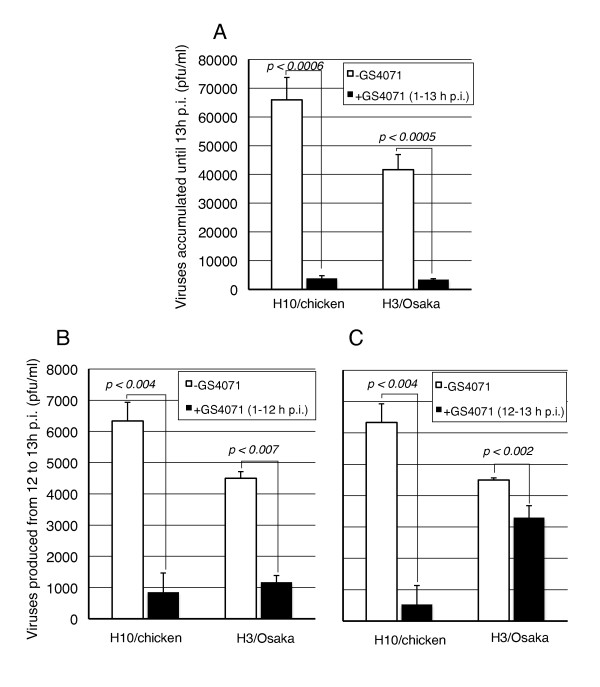
**Effects of GS4071 on the production of infectious viruses**. (A) Progeny viruses accumulated in the medium (assayed at 13 h p.i.). +GS4071: Virus-infected cultures were incubated with or without 2 μM GS4071 from 1 to 13 h p.i. H3/Osaka: human A/Osaka/981/98(H3N2). H10/chick: avian A/chicken/Germany/N/49(H10N7). Error bars represent standard deviations of averages of three independent experiments. (B) Progeny viruses produced from 12 to 13 h p.i. in the absence of GS4071. The virus-infected cell cultures were incubated with or without 2 μM GS4071 from 1 to 12 h p.i., washed to remove GS4071, and incubated in pre-warmed fresh DMEM from 12 to 13 h p.i. (for 1 h) at 36°C in the absence of GS4071. The culture medium was collected and assayed for the virus production. (C) Effect of pulse treatment with GS4071 on infected cells during virus production assay. Infected cells were incubated without GS4071 from 1 to 12 h p.i., and then in fresh medium with or without 2 μM GS4071 from 12 to 13 h p.i. (for 1 h) at 36°C. The culture medium was collected and analyzed for virus production. Statistical analyses were performed using the Student's *t*-test.

At 13 h p.i., the culture medium was collected to assay infectivity of progeny viruses. As shown in Figure 1A, incubation with GS4071 decreased virus production. However, the possibility still remained that progeny viruses could not be released from the surface of the infected cells because NA function was blocked by GS4071. To examine this possibility, cells were incubated with or without GS4071 from 1 to 12 h p.i., and then without GS4071 from 12 to 13 h p.i. As shown in Figure 1B, even in the absence of GS4071, virus production was poor when the culture was pretreated with the NA inhibitor. Thus it is possible that GS4071 directly decreased virus production.

In the next experiment, infected cells were pulse-treated with 2 μM GS4071 from 12 to 13 h p.i., and viruses produced during pulse treatment were assayed. As shown in Figure 1C, pulse treatment with GS4071 from 12 to 13 h p.i. affected evidently H10/chicken virus production, but not so H3/Osaka virus production. The same results as above were obtained when another NA inhibitor, 2 μM zanamivir (GG167), was used (data not shown).

### Effect of GS4071 on viral protein synthesis in infected cells

**Figure 2:**
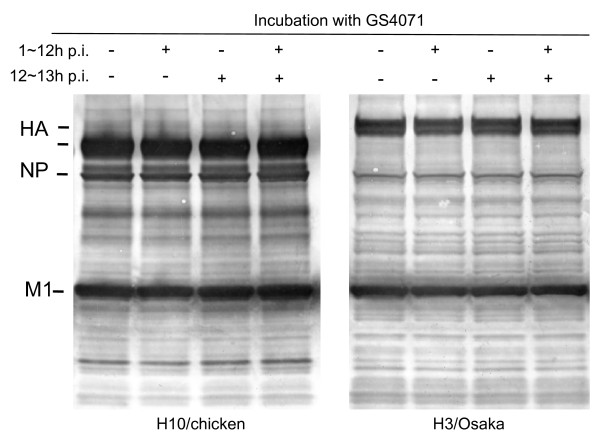
**Effect of GS4071 on viral protein accumulation in infected cells and viral particles**. MDCK cells infected with H10/chicken or H3/Osaka virus were incubated from 1 to 13 h p.i. with or without 2 μM GS4071, and the total amount of viral proteins in the cells and in the medium was analyzed with SDS-PAGE and western blotting.

To analyze which step of virus production was inhibited by GS4071, we examined the effect of GS4071 on viral protein synthesis in infected cells. Virus-infected cells were incubated with or without 2 μM GS4071 from 1 to 13 h p.i., and added to a protein lysis buffer for sodium dodecyl sulfate-polyacrylamide gel electrophoresis (SDS-PAGE). To assess the total amount of viral proteins accumulated in cells and virus particles, the culture medium at 13 h p.i. was centrifuged to sediment virus particles; lysis buffer was added to the precipitated viral particles, and this aliquot was mixed with the cell lysate. The total proteins accumulated were analyzed by SDS-PAGE and western blotting with anti-influenza A virus antibody. As shown in Figure 2, no significant difference was observed in the amount of viral proteins accumulated between cell cultures incubated with or without GS4071. This result indicates that GS4071 treatment does not cause any detectable decrease in viral protein synthesis under conditions used in this study. It is possible that the NA inhibitor interferes with the final step of viral morphogenesis, namely, virus particle formation.

### Scanning electron microscope (SEM) observation of the surface of infected cells

**Figure 3:**
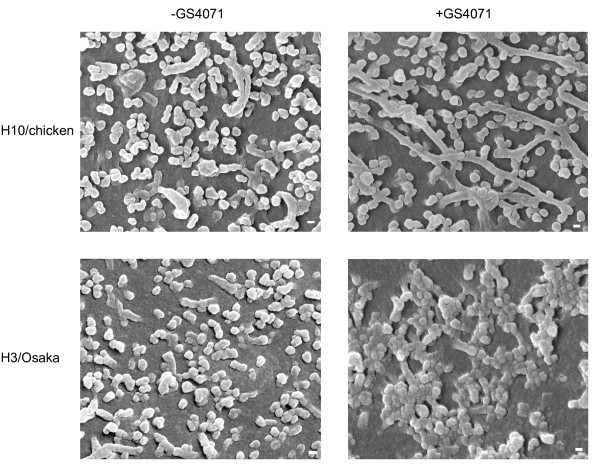
**Surface of the virus-infected cells observed with SEM**. MDCK cells infected with H10/chicken or H3/Osaka virus were incubated with or without 2 μM GS4071 from 1 to 13 h p.i., and then observed by SEM. Scale bar: 100 nm. Magnification: 30,000×.

To investigate the inhibitory mechanism of GS4071 against virus particle formation, the surface of infected cells was observed by SEM. MDCK cells infected with the viruses were incubated with or without GS4071 as described above. The cells were fixed and observed by SEM. As shown in Figure 3, many spherical buds (probably budding viruses) were observed on the surface of H3/Osaka virus-infected cells incubated without GS4071. However, when H3/Osaka virus-infected cells were incubated with 2 μM GS4071 from 1 to 13 h p.i., many particles aggregated with each other on the surface, but no single bud or particle was observed. This result was not beyond our expectations, because it is known that without NA activity, terminal sialic acid remains on the viral glycoprotein, and thereby causes self-aggregation of viruses through binding of HA to the sialoglycan [8-10]. On the other hand, when the H10/chicken virus-infected cells were incubated with GS4071, many long tubular viral-like structures and single buds were observed on the cell surface, but no aggregated particles were observed. It is possible that the single buds are immature viruses in the process of budding. The same result was obtained with another NA inhibitor (2 μM GG167) (data not shown).

Considering these results, it is highly possible that the NA inhibitor did not suppress viral replication until initiation of the budding process; thus, interfering with the process from budding to mature virus particle formation in H10/chicken virus-infected cells.

## Discussion

When H10/chicken-infected cells were incubated with GS4071, production of infectious particles clearly decreased (Figure 1A-C) and many long tubular projections were formed (Figure 3), indicating that GS4071 inhibited the formation of spherical virus particles (i.e., physical particles). On the other hand, when H3/Osaka-infected cells were incubated with GS4071, many aggregated particles were observed on the cell surface (Figure 3), indicating that physical particles were produced even in the presence of GS4071. Thus, we believe that the NA inhibitor has the potential to interfere with virus particle formation in H10/chicken virus-infected cells, but not in H3/Osaka virus-infected cells. Because various observations regarding NA function have been reported [1-12], which have not necessarily been consistent with each other, the effect of NA on viral entry, replication, assembly, and budding may not be uniform among various virus strains.

Although pulse treatment of H3/Osaka-infected cells with GS4071 was not as effective as that of H10/chicken-infected cells (Figure 1C), production of infectious viruses decreased with a significant difference (*p *< 0.002). Probably, the release of viruses was partially blocked by pulse treatment for 1 h, and approximately 20% progeny viruses remained on the cell surface as aggregates (Figure 1C). When treatment is prolonged for 12 h, the aggregated viruses may gradually accumulate and cover the cell surface (Figure 3), and thereby interfere with the release of progeny virus more efficiently.

**Figure 4:**
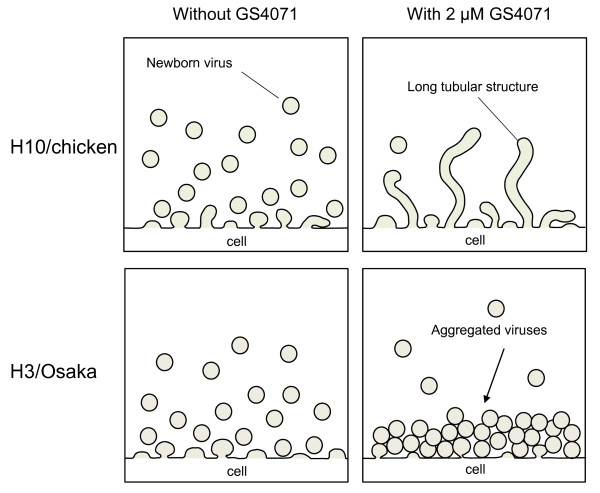
**Schematic illustration of the effect of the NA inhibitor on the production of progeny virus**. MDCK cells infected with H10/chicken or H3/Osaka virus were incubated with or without 2 μM GS4071 from 1 to 13 h p.i.

Based on our results, we have summarized the effect of the NA inhibitor on virus particle formation in a schematic illustration (Figure 4). In the absence of NA inhibitor, most mature virus particles are released and only immature viruses budding from the cell surface are observed as buds or projections in SEM (Figure 4, H3/Osaka and H10/chicken, -GS4071). When H3/Osaka virus-infected cells were incubated with the NA inhibitor, a considerable amount of aggregated particles, but no single particle or bud, were observed (Figure 4, H3/Osaka, +2 μM GS4071). However, when H10/chicken virus-infected cells were incubated with the NA inhibitor, no aggregated particles, but many long tubular-structures and single buds were observed on the cell surface (Figure 4, H10/chicken, +2 μM GS4071). Thus, the NA inhibitor interferes with the process from progression of budding to mature virus particle formation.

Considering the mechanism of virus particle formation, through morphological and functional analyses of virus-like particles (VLPs), viral matrix proteins have been believed to play an essential role in the budding process [13-15]. Recently, however, a study using a plasmid-derived expression system showed that HA and NA proteins were the minimal requirements for VLP formation [16]. Moreover, Lai et al. [17] reported that NA expression alone was sufficient to generate and release VLPs, and concluded that NA plays a key role in virus budding and morphogenesis. The above-cited studies do not necessarily report the same views on viral morphogenesis. This may be because of different virus strains used in the experiments. In the present study, the contribution of NA to viral morphogenesis appeared different between the two strains used in the experiment.

We attempted to abolish the effect of the NA inhibitor on H10/chicken-infected cells by adding *Vibrio cholerae *NA (VCNA) to the culture medium, but the inhibitory effect of GS4071 on virus production was not affected by VCNA (data not shown). This result indicates that viral NA activity is essential for virus particle formation in H10/chicken virus, and VCNA could not substitute viral NA in this case.

We found the novel function of the NA inhibitor against H10/chicken virus, so to say, by chance. Now we are interested in whether this effect of the NA inhibitor is restricted to H10/chicken virus or observed in other influenza virus strains. We have recently obtained a preliminary result indicating that the same effect is observed in avian influenza A/duck/Hokkaido/07(H10N2), A/duck/Hokkaido/00(H10N4) and A/duck/Hokkaido/06(H10N8) strains (data not shown). Further detailed experiments and analysis of the mechanism are being planned at our laboratory.

## Conclusions

This study demonstrates that NA inhibitors interfere with the final step of virus particle formation in H10/chicken virus-infected cells, suggesting that viral NA assists viral morphogenesis of H10/chicken virus.

## Methods

### Cells, viruses, and NA inhibitors

MDCK cells provided by Dr. T. Kase (Osaka Prefectural Institute of Public Health, Japan) were cultured in Eagle's minimum essential medium supplemented with 10% fetal calf serum (FCS). For viral infection, MDCK cells were washed twice with FCS-free medium and then supplemented with fresh DMEM.

The H10/chicken virus was provided by Dr. H. Kida (Hokkaido University, Japan) and the H3/Osaka virus Dr. Y. Okuno (Osaka Prefectural Institute of Public Health). All viral experiments were performed in a level 2 biosafety laboratory.

GS4071 and GG167 were obtained from Roche Products Ltd. (Welwyn, UK) and Glaxo Research and Development Ltd. (Stevenage, UK), respectively.

### Effect of GS4071 on the production and release of infectious viruses

Confluent monolayer cultures of MDCK in 6-well plates (Sumitomo Bakelite, Japan) were inoculated with H3/Osaka or H10/chicken virus at MOI of 0.3 pfu/cell and incubated for 1 h at 36°C, washed once with DMEM, and incubated further for 12 h (that is, from 1 to 13 h p.i.) at 36°C with or without 2 μM GS4071. To assay the infectivity of produced viruses, the culture medium was harvested. For accurate comparison of the virus yields in the presence and absence of GS4071, an equal concentration of GS4071 must be present in all samples, since this NA inhibitor is known to affect viral infection efficiency at the initial stage of infection [6]. Thus, GS4071 was added to the culture medium harvested in the absence of the NA inhibitor, in order to adjust the concentration immediately before the infectivity assay. The NA inhibitor concentration used in the present study was on the basis of that obtained in our previous study [6].

### Plaque titration assay

The harvested culture medium was serially diluted after adjustment of GS4071 concentration. MDCK cells prepared in a 12-well culture plate were inoculated with diluted viral suspensions and incubated for 1 h at 36°C, and then the culture medium was replaced with DMEM containing 0.6% agarose and 1 μg/ml acetylated trypsin (Sigma, USA). Two days after viral inoculation, plaques were stained with anti-H3/Osaka or anti-H10/chicken guinea pig antiserum (prepared in our laboratory) and peroxidase-conjugated goat anti-guinea pig IgG (Jackson ImmunoResearch, USA). Antibody binding was detected by 3,3'-diaminobenzidine (Sigma, USA) staining.

### Detection of viral proteins accumulated in virus-infected cells and virus particles in the absence or presence of GS4071

MDCK cells were infected with H10/chicken or H3/Osaka virus and incubated with or without 2 μM GS4071 from 1 to 13 h p.i. Cells were lysed with SDS-PAGE lysis buffer (ATTO, Japan), and the virus particles released in the culture medium were collected by centrifugation at 39,000 ×*g *for 180 min at 4°C; this was followed by addition of lysis buffer to the precipitated viruses. Both lysates were then combined to evaluate the total amount of viral proteins accumulated and centrifuged at 20,000 ×*g *for 60 min at 4°C to remove insoluble materials. The supernatant was analyzed by SDS-PAGE and western blotting. Viral proteins were treated with anti-H3/Osaka or anti-H10/chicken guinea pig antiserum and peroxidase-conjugated goat anti-guinea pig IgG, and detected by 3,3', 5,5'-tetramethylbenzidine (ATTO) staining.

### SEM observation of the surface of infected cells

Confluent monolayer culture of MDCK cells prepared on a poly-L-lysine-coated cover glass with a diameter of 12 mm (Asahi Glass Co., Japan) was inoculated with the virus and incubated at 36°C for 1 h. At 1 h p.i., the culture was washed twice with DMEM to remove free viruses and incubated at 36°C from 1 to 13 h p.i. with or without 2 μM GS4071. The cells were fixed with 2.5% glutaraldehyde in 0.15 M phosphate buffer (pH 7.4) and post-fixed with

1% osmium tetroxide. The fixed cells were dehydrated through a series of ethanol and butanol mixtures [18], and observed under the JSM-6340 SEM (JEOL, Japan).

## Competing interests

The authors declare that they have no competing interests.

## Authors' contributions

HU performed the infectivity assay, analysis of viral proteins, SEM observations, and data analysis. MO designed the study, analyzed data, and drafted the manuscript. All authors read and approved the final manuscript.
